# Comparison of three different types of exercises for selective contractions of supra- and infrahyoid muscles

**DOI:** 10.1038/s41598-021-86502-w

**Published:** 2021-03-30

**Authors:** Min Cheol Chang, Sungwon Park, Joo Young Cho, Byung Joo Lee, Jong-Moon Hwang, KwanMyung Kim, Donghwi Park

**Affiliations:** 1 Department of Rehabilitation Medicine, Yeungnam University Hospital, Daegu, Republic of Korea; 2 Department of Rehabilitation Medicine, Daegu Fatima Hospital, Daegu, Republic of Korea; 3 Department of Rehabilitation Medicine, School of Medicine, Kyungpook National University, Kyungpook National University Hospital, Daegu, Republic of Korea; 4 Graduate School of Creative Design Engineering, Ulsan National Institute of Science and Technology, Ulsan, Republic of Korea; 5 Department of Physical Medicine and Rehabilitation, Ulsan University Hospital, University of Ulsan College of Medicine, 877, Bangeojinsunhwando-ro, Dong-gu, Ulsan, 44033 Republic of Korea

**Keywords:** Neurological disorders, Geriatrics, Quality of life

## Abstract

Several exercise methods, such as the Shaker exercise, tongue press exercise, chin tuck against resistance (CTAR) exercise, and submandibular push exercise, have been introduced to strengthen the muscles involved in swallowing. In this study, we compared the effectiveness of the CTAR, submandibular push, and Shaker exercises for the induction of selective supra- and infrahyoid muscle contractions using surface electromyography (EMG). This study is a prospective non-randomized controlled study. Twenty-five healthy subjects and 20 patients experiencing swallowing difficulty were enrolled. During the three different types of exercises, the root mean square (RMS) values of the sternocleidomastoid (SCM), suprahyoid (anterior belly of the digastric and mylohyoid muscles), and infrahyoid (sternothyroid and thyrohyoid muscles) muscles were analyzed using surface EMG. Differences in the activity of swallowing muscles among the three different exercises were analyzed using one-way repeated measured analysis of variance. In terms of both the maximum and mean RMS values of the suprahyoid muscle, the submandibular push exercise showed a larger RMS value than the CTAR and Shaker exercises in healthy subjects (*p* < 0.05). In terms of both the maximum and mean RMS values of the suprahyoid muscle, the Shaker exercise and submandibular push exercise showed a larger RMS value than the CTAR exercise in patients with swallowing difficulty (*p* < 0.05). The submandibular push exercise may be effective as a swallowing muscle exercise owing to its superiority in inducing selective contractions of the supra- and infrahyoid muscles. The CTAR and Shaker exercises are also effective in this regard.

## Introduction

The swallowing process is a compound sensori-motor behavior that includes the coordinated relaxation and contraction of the muscle tissues around the tongue, mouth, pharynx, larynx, and upper esophagus^1–3^. Of the muscles contributing to the swallowing process, the supra- and infrahyoid muscles have been extensively reported^2,4–7^. The functions of the suprahyoid muscle in terms of the anterosuperior motions of the hyoid bone and the functions of the omohyoid, sternothyroid, and sternohyoid muscles and a part of the infrahyoid muscles in depressing the hyolaryngeal complex are understood^8,9^. The role of the thyrohyoid muscle, one of the four infrahyoid muscles, is to move the larynx antero-superiorly during the swallowing process. Moreover, the infra- and suprahyoid muscles support the opening of the upper esophageal sphincter (UES) by moving the hyoid bone anteriorly^8–12^.

Dysphagia or difficulty swallowing may result from various problems such as neurological impairment and physiological and anatomical derangements in any region from the mouth to the esophagus (e.g., in the muscles involved in swallowing)^13–17^. Various methods have been employed to treat dysphagia. Conventional treatment methods such as oropharyngeal (including the muscles involved in swallowing) exercises, compensated maneuvers, and electrical stimulation of the oropharyngeal area are widely applied in swallowing therapy^11,18–21^.

The muscles involved in swallowing can be strengthened using several exercise methods, such as the Shaker and tongue press exercises, chin tuck against resistance (CTAR) exercise, and submandibular push exercise^20,21^. In the Shaker exercise, sustained head lifts are performed with the subject in the supine position. The Shaker exercise was designed to strengthen the suprahyoid muscle, thereby assisting the opening of the UES. It can be effective in addressing swallowing difficulty caused by incomplete UES opening^20^. Additionally, the Shaker exercise significantly increases the antero-posterior diameter of the UES in elderly patients with and without swallowing difficulty and significantly reduces post-swallow aspiration^20^. However, in a previous study, it was concluded that the Shaker exercise was difficult to perform, especially for elderly patients with chronic diseases^22^. Hence, the CTAR exercise was recommended in previous studies^21,23^. In the CTAR exercise, by changing the resistances of a plastic bar or rubber ball between the chest and the chin, the exercise load can be modulated^21,23^. However, the superiority of the CTAR exercise over the Shaker exercise has been disputed. In a previous study, the CTAR exercise showed greater maximal values on surface electromyography (EMG) of the suprahyoid muscle than the Shaker exercise^23^. However, in patients with swallowing difficulty, Gao and Zhang^24^ reported similar effects in improving swallowing dysfunction between the CTAR and Shaker exercises.

However, both exercises involve neck flexion movement patterns, which induce unnecessary contractions of the sternocleidomastoid (SCM) muscle and may adversely affect contractions of the selective supra- and infrahyoid muscles^25^. To overcome the limitations of the CTAR and Shaker exercises, the submandibular push exercise was introduced in a previous study^18^. However, that study used visual feedback from pressure sensors. Therefore, the application of the submandibular push exercises in patients who constantly need dysphagia treatment may have some limitations. Based on these observations, we examined the effectiveness of the submandibular push exercise for the contraction of muscles involved in swallowing without a pressure sensor, in order to determine its usefulness as an at-home exercise. Based on previous research results, we postulated that compared to the CTAR and Shaker exercises, the submandibular push exercise would induce selective contraction of the supra- and infrahyoid muscles without unnecessary SCM contraction. Therefore, we assessed the effectiveness of the CTAR, submandibular push, and Shaker exercises for the induction of selective contractions of the supra- and infrahyoid muscles using a simple device, such as a plastic bar. By comparing three different exercises, we attempted to determine the effects of each of the three different exercises on each swallowing muscle in healthy subjects and patients with swallowing difficulty.

## Methods

### Ethics statements

This prospective case–control study was approved by the institutional review board of Daegu Fatima Hospital. All methods were performed in accordance with relevant guidelines and regulations (Declaration of Helsinki), and written informed consent was obtained from all participants. Informed consent for publication of identifying images was obtained from each relevant patient.

### Participants

In this study, 45 participants (25 healthy participants and 20 patients with swallowing difficulty) were enrolled (Table 1). The healthy subjects were participants without swallowing difficulty or any history of diseases that could cause swallowing difficulty (such as traumatic brain injury, spinal cord injury, and stroke). Patients with swallowing difficulty had various causative reasons and exhibited at least one symptom of swallowing difficulty, such as coughing when eating, a wet or weak voice, food sticking in the throat, globus sensations, difficulty in chewing, and drooling^26,27^. We excluded patients with problems that prevented them from holding an exercise tool during exercise, those with severe cognitive dysfunction (Mini-Mental Status Examination score ≤ 9), those with serious psychiatric disorders, and those aged < 20 years.

**Table 1:** Characteristics of participants.

	Healthy participants	Patients with swallowing difficulty
Number	25	20
Sex ratio (M:F)	21:4	12:8
Age (years)	29.9 ± 4.1	70.6 ± 9.0
Duration (months)		6.2 ± 4.5
Cause of dysphagia		Hemispheric stroke (n = 14)
		(SICH = 1, infarction = 13)
		brainstem stroke (n = 5)
		(SICH = 1, infarction = 4)
		TSAH (n = 1)
Symptoms of dysphagia		Protective cough with eating (n = 20)
		Food sticking in throat (n = 10)
		Drooling (n = 3)
		Having a wet or weak voice (n = 6)
		Globus sensation (n = 10)
		Difficulty chewing (n = 7)

Mean ± standard deviation, M:F; male:female, TSAH; traumatic subarachnoid hemorrhage, SICH; spontaneous intra-cerebral hemorrhage.

### Design

All participants were asked to consecutively perform three different types of exercises (submandibular push, CTAR, and Shaker exercises). Participants were permitted to rest between each exercise session, to prevent muscle fatigue. Before the start of each exercise, the resting muscle (supra- and infrahyoid muscles) activation levels were recorded using surface EMG.

### Procedures

All participants were assessed in a quiet room. Each participant was asked to perform one trial of each exercise (10 repetitions in each trial) in the following order: (1) the Shaker exercise, (2) the CTAR exercise, and (3) the submandibular push exercise, with a 10-min rest between each exercise session to prevent fatigue of the swallowing muscles. For each exercise, the participants were instructed to maintain the end-position for 10 s^21,23^. The participants were instructed to keep their mouths closed during all the exercises.

### Exercise maneuvers

**Figure 1:**
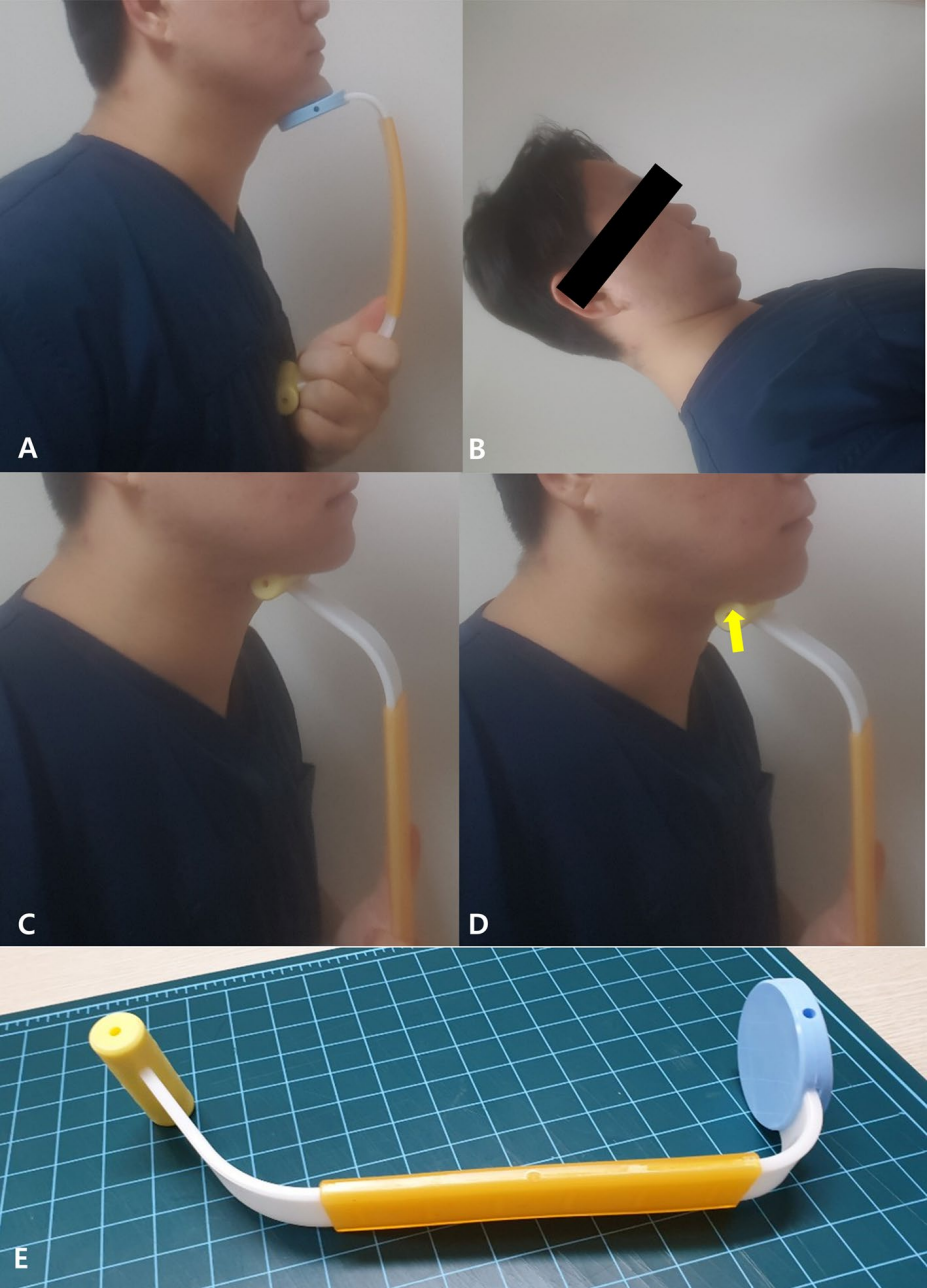
(**A**) Chin tuck against resistance (CTAR) exercise. (**B**) Shaker exercise. (**C**, **D**) Submandibular push exercise. Arrow, bloating change in the submandibular area during the submandibular push exercise (**E**) A plastic device used in this study.

First, when performing the Shaker exercise, the healthy subjects and patients were instructed to perform the following exercise protocol: lie on the bed in the supine position, lift the head up and hold it for 10 s (Fig. 1A)^18,21,23,24^. Second, when performing the CTAR exercise, participants were instructed to sit on a chair and press the chin as hard as possible against a CTAR device (Renodevice, Daegu, South Korea) placed under the chin (Fig. 1B) ^21,23^. Lastly, when performing the submandibular push exercise, the participants were instructed to sit on a chair and increase the pressure at the submandibular area against a plastic device [Renodevice, Daegu, South Korea, (Fig. 1C,D)] without neck flexion^18^. The participants were instructed to maintain the end posture during the three exercises, and the surface EMG data were recorded.

### Electromyography

**Figure 2:**
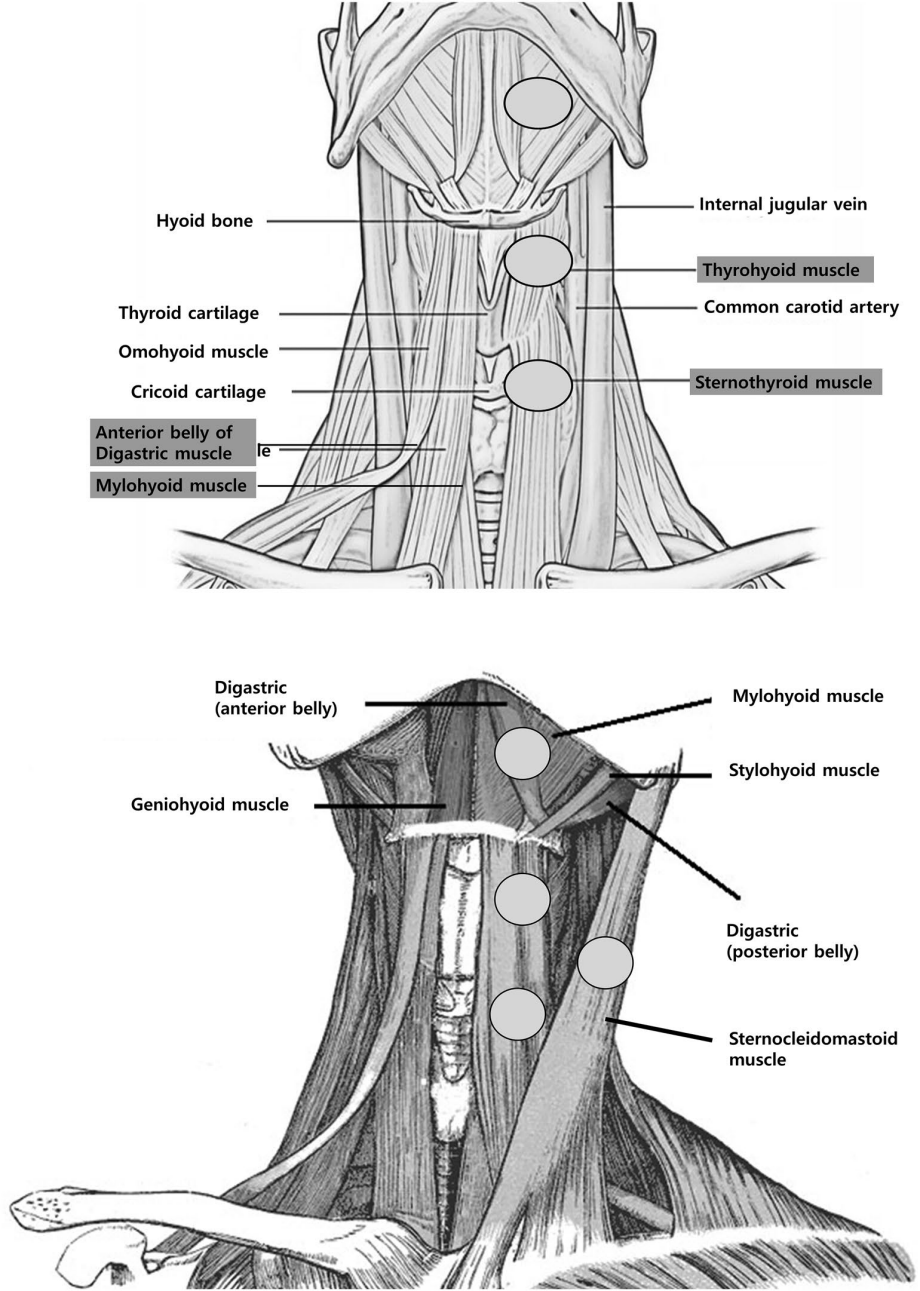
Locations of the surface electromyography electrodes during the chin tuck against resistance, Shaker, and submandibular push exercises.

Using an EMG device and software (Medelec Synergy; CareFusion Corporation, San Diego, CA), multi-channel surface EMG was performed during each exercise^2,28^. The surface EMG electrode was attached to the SCM, suprahyoid muscles (mylohyoid and anterior belly of the digastric muscles), and infrahyoid muscles (sternothyroid and thyrohyoid muscles) (Fig. 2)^2^. To confirm the precise location of the surface EMG electrodes on the targeted muscles, ultrasonographic examinations were performed before the attachment of surface EMG electrodes. The location of the bellies of the suprahyoid, infrahyoid, and SCM muscles was examined using a 3–16- MHz linear ultrasound probe (Samsung Medison, Hongchun-gun, South Korea)^2^. The reference surface electrode was positioned on the anterior surface of the clavicle or mandible bone, the active surface electrode on the belly of the examined muscle, and the ground electrodes on the mandible base. To accommodate the surface EMG, all participants rested for 10 min before each exercise. During exercises involving a stick, an attempt was made to prevent contact between the stick and surface EMG electrode to reduce artifacts.

Data were collected at a sampling rate of 50 kHz using Medelec Synergy with the following settings: high-frequency filter, 1000 Hz; low-frequency filter, 10 Hz; 1 s/div and 100 μV/div; sweep speed and gain; and common mode rejection ratio, > 110 dB^2,28,29^. The EMG activity was measured using a custom-designed disposable pre-gelled 20-mm Ag/AgCl disc electrode (CareFusion, Höchberg, Germany)^18^.

### Signal processing

The root mean square (RMS) value of EMG activity was measured 10 times with 1 s as a section^18^. For 10 s, the patient maintained the end-position of each exercise, and the RMS value (during 1 s as a section) was measured 10 times consecutively. Using EMG software (Medelec Synergy; CareFusion Corporation), the mean RMS of the EMG activity of each swallowing muscle during 1 s was calculated. In each exercise maneuver, a mean RMS value during 10 s was calculated^18^.

The resting RMS value was subtracted from the RMS value measured for each muscle to minimize bias caused by muscle contraction due to posture maintenance before exercise. Although this method is not a commonly used normalization of the EMG signal method and as it is difficult to induce maximum contraction of the swallowing muscles in patients, we used the resting RMS value of each target muscle according to the exercise posture before starting the exercise. We attempted to minimize the bias by subtracting it from the maximum RMS value of each target muscle.

The highest RMS value was defined as the maximum RMS value among the 10 RMS values (1-s section) measured for each exercise maneuver^18^. In addition, the average RMS value was calculated as the average of the 10 RMS values (1-s section)^18^.

### Statistical analyses

PRISM version 8.00 (GraphPad Software, Inc., San Diego, CA, USA) and IBM SPSS version 21 (SPSS, Inc., Chicago, IL, USA) were used for all statistical analyses. Differences in the activity of the swallowing muscles among the three different exercises were analyzed using one-way repeated measured analysis of variance (ANOVA). Using repeated-measures ANOVA with the Tukey post-hoc test, the maximum and average RMS values of the 35 participants were then compared. A *p* value < 0.05 was considered statistically significant.

### Sample size calculation

The maximum RMS value of the suprahyoid muscle (μV) was determined using data from the 10 healthy subjects. This value was used to perform a two-tailed sample size calculation of the expected mean differences, with a standard deviation (SD) of 0.2, significance level of 0.05, and power of 80%. Therefore, the required sample size for this study was at least 12 participants in each group.

## Results

### Participant characteristics

Twenty-five healthy participants (male: 21, female: 4) aged 21–39 years (average, 29.9; SD, 4.1) and 20 patients with swallowing difficulty (male: 12, female: 8) aged 52–84 years (mean, 70.6; SD, 9.0) were enrolled (Table 1). The etiologies of swallowing difficulty are shown in Table 1.

### Comparison of the three different types of exercises in the healthy subjects

**Figure 3:**
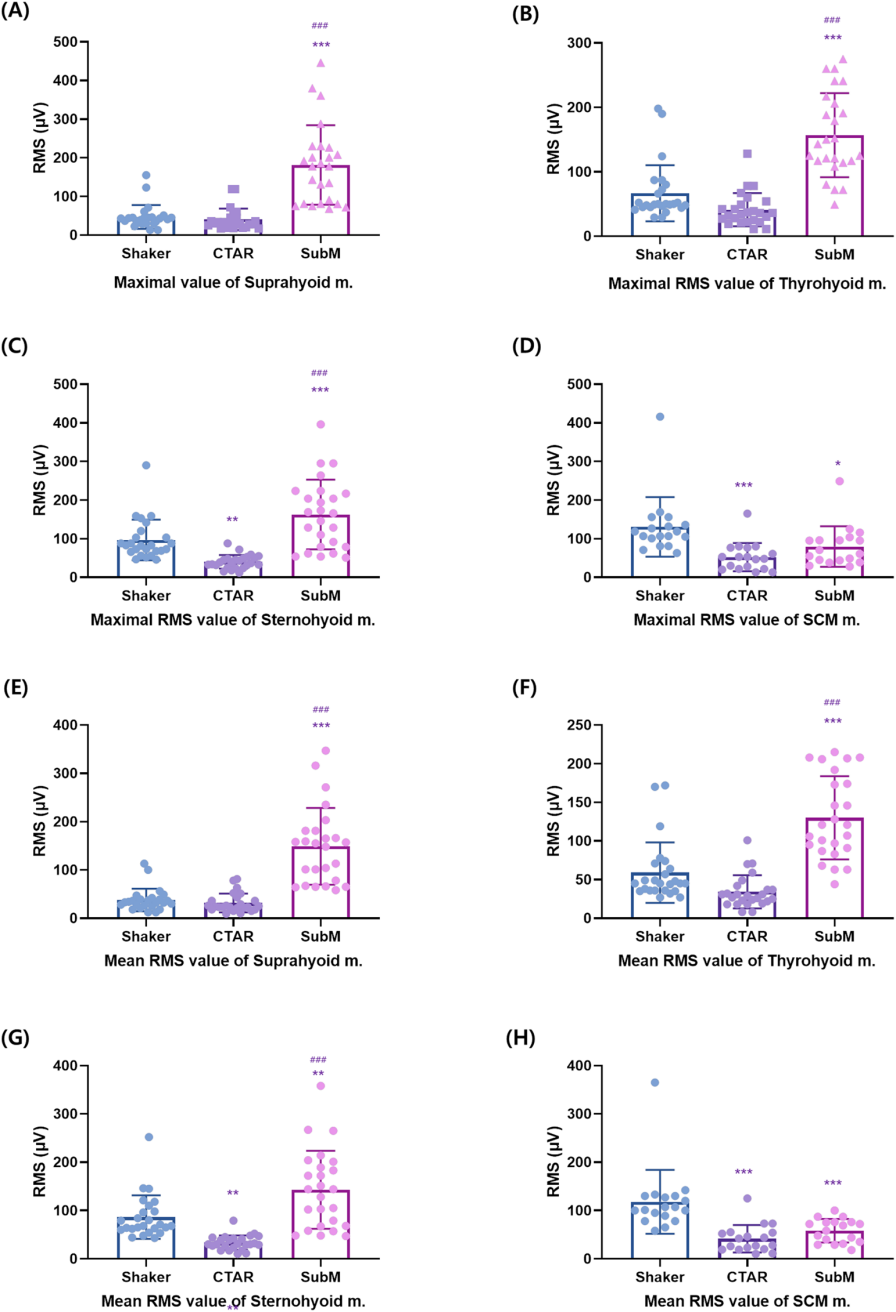
The maximum (**A**–**D**) and mean (**E**–**H**) RMS values of the suprahyoid, thyrohyoid, sternohyoid, and SCM muscles in healthy participants. The graphs were drawn using GraphPad Prism 8.0. RMS, root mean square; SCM, sternocleidomastoid. Compared with the Shaker exercise: *p < 0.05, **p < 0.01, ***p < 0.001; compared with the chin tuck against resistance exercise: ^#^*p* < 0.05, ^##^*p* < 0.01, ^###^*p* < 0.001.

There were significant differences in the maximum RMS values of the suprahyoid (*p* < 0.001), thyrohyoid (*p* = 0.003), sternohyoid (*p* < 0.001), and SCM muscles (*p* < 0.001) among the three different exercises, as well as in the mean RMS values of the suprahyoid (*p* < 0.001), thyrohyoid (*p* < 0.001), sternohyoid (*p* < 0.001), and SCM muscles (*p* < 0.001). In the post-hoc analysis, the submandibular push exercise exhibited a larger RMS value than the Shaker and CTAR exercises in terms of both the maximum and mean RMS values of the suprahyoid muscle (*p* < 0.05, Tables 2, 3). There was no significant difference between the CTAR and Shaker exercises (*p* ≥ 0.05). In terms of both the maximum and mean RMS values of the thyrohyoid muscle, the submandibular exercise exhibited a larger RMS value than the Shaker and CTAR exercises (*p* < 0.05). However, there was no significant difference (*p* ≥ 0.05) between the Shaker and CTAR exercises. In terms of both the maximum and mean RMS values of the sternohyoid muscle, the submandibular push exercise showed a larger RMS value than the Shaker and CTAR exercises (*p* < 0.05). Moreover, between the CTAR and Shaker exercises, there were significant differences (*p* < 0.05). In terms of both the maximum and mean RMS values of the SCM muscle, the Shaker exercise showed a larger RMS value than the CTAR and submandibular push exercises (*p* < 0.001). However, there was no significant difference between the CTAR and submandibular push exercises (*p* < 0.05) (Fig. 3A–H).

**Table 2:** The maximal RMS value of the suprahyoid, thyrohyoid, sternohyoid, and SCM muscles in healthy participants and patients with dysphagia.

		Max Suprahyoid RMS			Max Thyrohyoid RMS			Max Sternothyroid RMS			Max SCM RMS		
		Shaker (μV)	CTAR (μV)	SubM (μV)	Shaker (μV)	CTAR (μV)	SubM (μV)	Shaker (μV)	CTAR (μV)	SubM (μV)	Shaker (μV)	CTAR (μV)	SubM (μV)
HP	Mean	47.04	40.64	181.52*^#^	66.76	41.12	156.96*^#^	96.80^#^	40.20*	162.88*^#^	130.72^#^	52.33*	79.78*
	SD	30.79	27.73	102.83	43.58	25.76	65.28	52.58	17.45	90.24	76.99	36.49	52.37
DP	Mean	78.95^#^	35.61*	76.24^#^	100.50^#^	43.39*	69.74^#^	95.85^#^	37.07*	76.87^#^	98.66^#^	53.64*	54.80*
	SD	43.35	26.07	36.15	61.19	32.51	54.49	25.52	26.59	42.98	34.48	28.17	26.61

RMS; root mean square, SD; standard deviation, CTAR; chin tuck against resistance, SubM; submandibular push exercise, SCM; sternocleidomastoid, Max; maximum, HP; healthy participants, DP; dysphagic patients.

versus Shaker; *p < 0.05, versus CTAR; #*p* < 0.05.

**Table 3:** The mean RMS value of the suprahyoid, thyrohyoid, sternohyoid, and SCM muscles in healthy participants and patients with dysphagia.

		Mean Suprahyoid RMS			Mean Thyrohyoid RMS			Mean Sternothyroid RMS			Mean SCM RMS		
		Shaker (μV)	CTAR (μV)	SubM (μV)	Shaker (μV)	CTAR (μV)	SubM (μV)	Shaker (μV)	CTAR (μV)	SubM (μV)	Shaker (μV)	CTAR (μV)	SubM (μV)
HP	Mean	37.64	31.57	148.91*^#^	59.12	34.19	130.07*^#^	86.33^#^	33.42*	142.92*^#^	118.06^#^	41.39*	58.11*
	SD	23.43	19.43	79.41	39.02	21.43	53.86	45.22	15.06	80.76	66.26	28.53	24.28
DP	Mean	62.47^#^	30.05*	67.28^#^	83.31^#^	34.49*	60.30	86.32^#^	33.06*	67.62^#^	88.23^#^	42.47*	47.24*
	SD	28.27	19.58	35.44	34.86	21.57	46.58	22.67	23.67	45.37	29.64	19.6	20.86

RMS; root mean square, SD; standard deviation, CTAR; chin tuck against resistance, SubM; submandibular push exercise, SCM; sternocleidomastoid, Max; maximum, HP; healthy participants, DP; dysphagic patients.

versus Shaker; *p < 0.05, versus CTAR; #*p* < 0.05.

### Comparison of the three different types of exercises in patients with swallowing difficulty

There were significant differences in the maximum RMS values of the suprahyoid (*p* < 0.001), thyrohyoid (*p* < 0.001), sternohyoid (*p* < 0.001), and SCM muscles (*p* < 0.001) among the three different exercises, as well as the mean RMS values of the suprahyoid (*p* < 0.001), thyrohyoid (*p* < 0.001), sternohyoid (*p* < 0.001), and SCM muscles (*p* < 0.001). In the post-hoc analysis, the Shaker and submandibular push exercises showed a larger RMS value than the CTAR exercise in terms of both the maximum and mean RMS values of the suprahyoid muscle (*p* < 0.05, Tables 2, 3). However, there was no significant difference between the submandibular push exercise and Shaker exercises (*p* ≥ 0.05). In terms of both the maximum and mean RMS values of the thyrohyoid muscle, the Shaker exercise showed a larger RMS value than the CTAR exercise (*p* < 0.05). However, there was no significant difference between the submandibular push and Shaker exercises (*p* ≥ 0.05) and between the submandibular push and CTAR exercises (*p* ≥ 0.05).

**Figure 4:**
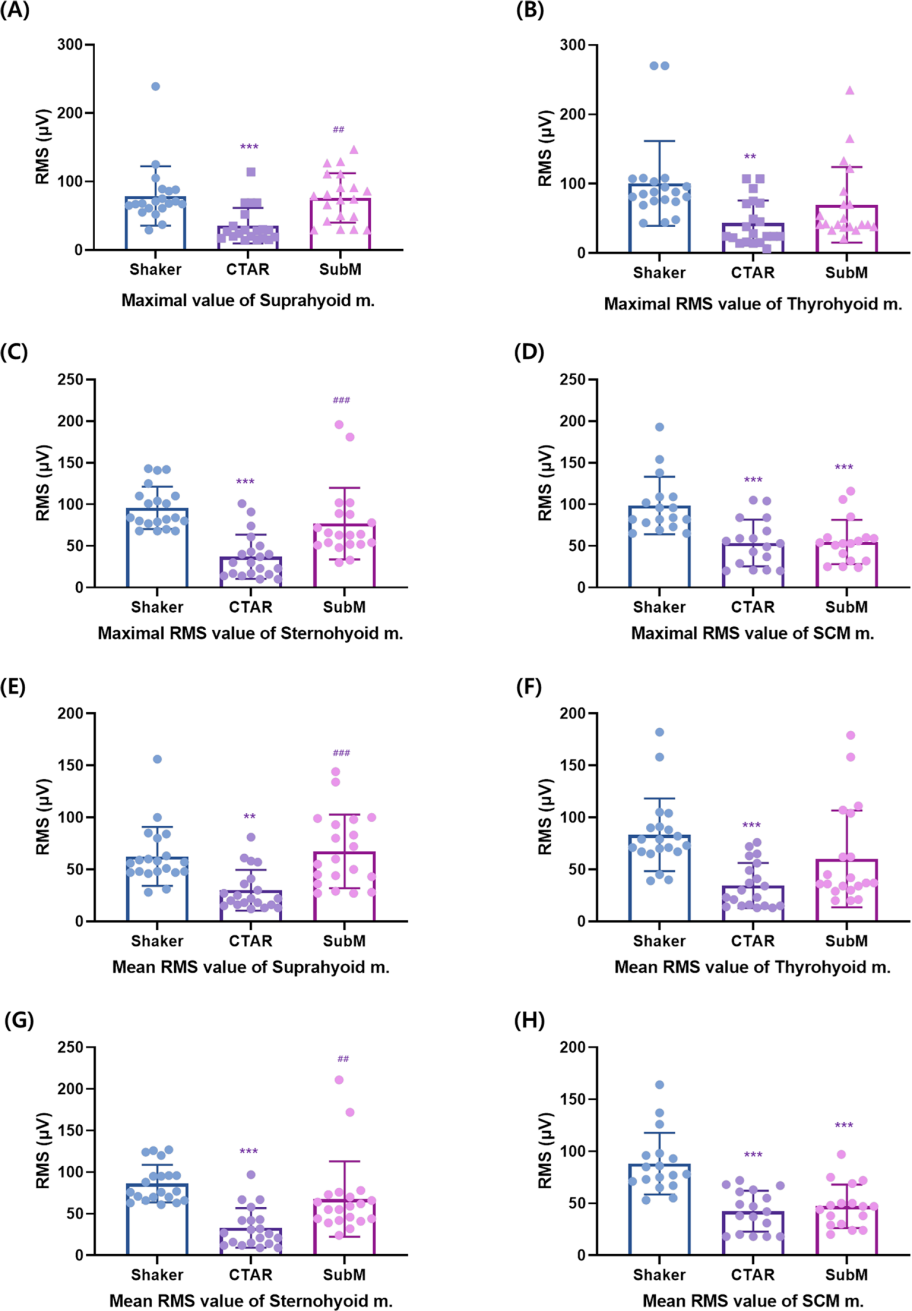
The maximum (**A**–**D**) and mean (**E**–**H**) RMS values of the suprahyoid, thyrohyoid, sternohyoid, and SCM muscles in patients with swallowing difficulty. The graphs were drawn using GraphPad Prism 8.0. RMS, root mean square; SCM, sternocleidomastoid. Compared with the Shaker exercise: *p < 0.05, **p < 0.01, ***p < 0.001; compared with the chin tuck against resistance exercise: ^#^*p* < 0.05, ^##^*p* < 0.01, ^###^*p* < 0.001.

In terms of both the maximum and mean RMS values of the sternohyoid muscle, the Shaker and submandibular push exercises showed a larger RMS value than the CTAR exercise (*p* < 0.05). However, there was no significant difference between the Shaker and submandibular push exercises, (*p* ≥ 0.05). In terms of both the maximum and mean RMS values of the SCM muscle, the Shaker exercise showed a larger RMS value than the CTAR and submandibular push exercises (*p* < 0.001). However, there was no significant difference (*p* < 0.05, Fig. 4A–H) between the CTAR and submandibular push exercises.

## Discussion

To the best of our knowledge, this is the first study to compare the effects of these three exercises (Shaker, CTAR, and submandibular push exercises) on the swallowing-related muscles using a plastic bar. The importance of this study is the knowledge of how each of the three different exercises affects each swallowing muscle in healthy subjects and patients with swallowing difficulty.

In the present study, both the maximum and mean RMS values of all the examined muscles showed significant differences according to the three different exercises in both healthy subjects and patients with swallowing difficulty. However, the muscle contraction patterns during the three different exercises showed slightly different results between healthy subjects and patients with swallowing difficulty. In the healthy young age group (20–40-year-old participants), the submandibular push exercise using a plastic bar induced stronger supra- and infrahyoid muscle contractions than the CTAR and Shaker exercises. The CTAR and Shaker exercises induced similar SCM, suprahyoid, and infrahyoid muscle contractions in healthy subjects, possibly because both these exercises included neck flexion movement patterns. The overall muscle contraction was higher in the Shaker exercise group than in the CTAR group. This is potentially because the Shaker exercise is performed with neck flexion against gravity.

In patients with swallowing difficulty, the degree of supra- and infrahyoid muscle contraction with the submandibular push exercise was higher than that with the CTAR exercise but similar to that with the Shaker exercise. However, in patients with swallowing difficulty, the submandibular push exercise exhibited less SCM muscle contraction than supra- and infrahyoid muscle contraction, unlike the other two exercise methods. The different contraction patterns of the swallowing muscles between healthy subject and patients with swallowing difficulty may be attributed to the following reasons: First, the difficulty in performing the submandibular push exercise. In a previous study, although the submandibular push exercise was performed with the help of visual feedback from a pressure sensor, patients experienced difficulty in understanding the submandibular push exercise^18^. Moreover, in our study, unlike healthy subjects who were in their 20 s and 30 s, the mean age of patients with dysphagia was 70 years; this discrepancy could be the cause of the difference in the effects of the submandibular push exercise between the two groups. Second, most patients with dysphagia often have weakness of the swallowing muscles such as the supra- and infrahyoid muscles, which decrease the degree of supra- and infrahyoid muscle contractions during the submandibular push exercise that requires a relatively high degree of swallowing muscle contraction^6^. However, in patients with swallowing difficulty, the submandibular push exercise along with the Shaker exercise induced the most supra- and infrahyoid muscle contractions.

Although the Shaker exercise can induce powerful contractions of muscles involved in swallowing, it may be inefficient from the perspective of selective contraction of muscles involved in swallowing (supra- and infrahyoid muscles)^30^. However, in patients with swallowing difficulty, the submandibular push exercise has been shown to induce powerful supra- and infrahyoid contractions similar to those induced by the Shaker exercise. In addition, in healthy subjects, the submandibular push exercise has been shown to induce more powerful supra- and infrahyoid muscle contractions than the Shaker exercise. Moreover, the submandibular push exercise can induce selective supra- and infrahyoid muscle contractions, considering the relatively lower degree of SCM muscle contraction. In a previous study, in terms of contraction of the SCM muscles in the CTAR and Shaker exercises, the CTAR exercise resulted in less activation of the SCM muscles than the Shaker exercise^22^. Although there may be a different degree of neck flexion movement, both the CTAR and Shaker exercises, unlike the submandibular push exercise, have a neck flexion component during exercise. These results demonstrate that the effects of the submandibular push exercise on both the supra- and infrahyoid muscle may be greater or equivalent to those of the CTAR or Shaker exercise without the induction of unnecessary SCM muscle contraction.

However, our results were inconsistent with those of some previous studies that reported the superiority of the CTAR exercise over other exercises such as the Shaker exercise with regard to the degree of suprahyoid muscle contraction^22,23^. This inconsistency may be due to the difference in devices used in the CTAR exercise. In previous investigations, soft balls were used in the CTAR exercise, whereas in our study, a plastic bar was used^22,23^. We thought that if devices with different elasticity were used in the CTAR and submandibular push exercises, this could create a bias in comparing the effects of the CTAR and submandibular push exercises. Therefore, to reduce bias due to differences in elasticity of the device, a plastic bar that can be used in both these exercises was applied to compare the effects of these two exercises.

### Limitations

There were several limitations in our study. First, we analyzed the degrees of supra- and infrahyoid muscle contractions in one session of each exercise. In addition, at least 2–3 months of training is necessary to cause functional changes in neuro-muscular tissue^31^. Functional changes such as the strengthening or thickening of muscle tissues in the supra- and infrahyoid muscles are difficult to prove, based on the results of this study. However, considering that the degree of muscle activation is proportional to the amount of exercise, the results of our study may reflect the possibility of actual strengthening of swallowing muscles. To confirm the improvement of dysphagia by the submandibular push exercise, a further study with > 2–3 months of exercise training is necessary^31^. Second, the order of execution of the three exercises was the same. To exclude bias caused by the order of exercise execution, future studies including a protocol with a random order of exercise execution would be necessary. Fourth, in this study, we used surface EMG; therefore, the effect of volume contraction cannot be completely excluded. In addition, artifacts due to the use of sticks may also affect the results of the three different exercises. Fifth, a standard EMG processing procedure was not used in this study. We calculated the RMS value of each muscle by subtracting the resting RMS value from the RMS value measured for each muscle. However, this study did not aim to compare the degree of contraction of several muscles according to each exercise. We only compared the RMS values to determine the activity induced by the three exercises in each muscle. For example, this study did not investigate the ratio of activation of the suprahyoid, infrahyoid, and SCM muscles with the three different exercises. We compared the RMS values of the Shaker, CTAR, and submandibular push exercises in one muscle of the same individuals. Therefore, although we did not use a standard EMG processing procedure, if we compare the degree of contraction in each participant using the same measurement method in one muscle, we believe that the results of this study. However, to investigate the exact RMS values of swallowing-related muscles, a further study with standard EMG processing would be required. Lastly, despite the use of surface EMG analysis, not all the muscles involved in swallowing were evaluated. Further studies investigating all the muscles involved in swallowing are warranted to investigate the exact mechanism and effects of the submandibular push exercise.

## Conclusions

Although the Shaker and CTAR exercises can induce powerful contractions of muscles involved in swallowing, they may be inefficient from the perspective of selective contraction of the supra- and infrahyoid muscles because of unnecessary contraction of the SCM muscle. Considering its superiority in inducing selective supra- and infrahyoid muscle contractions, the submandibular push exercise may be an effective exercise method in both healthy subjects and patients with swallowing difficulty. To confirm the improvement of dysphagia by using the submandibular push exercise, further studies with > 3 months of exercise training are warranted.
